# Functionalized Silica Star-Shaped Nanoparticles and Human Mesenchymal Stem Cells: An In Vitro Model

**DOI:** 10.3390/nano11030779

**Published:** 2021-03-18

**Authors:** Chiara Argentati, Francesco Morena, Chiara Fontana, Ilaria Tortorella, Carla Emiliani, Loredana Latterini, Giulia Zampini, Sabata Martino

**Affiliations:** 1 Department of Chemistry, Biology and Biotechnology, University of Perugia, Via del Giochetto, 06123 Perugia, Italy; chiara.argentati@unipg.it (C.A.); francesco.morena@unipg.it (F.M.); tortorellailaria@gmail.com (I.T.); carla.emiliani@unipg.it (C.E.); 2 Department of Chemistry, Biology and Biotechnology, University of Perugia, Via Elce di Sotto 8, 06123 Perugia, Italy; chiara.fontana@studenti.unipg.it (C.F.); loredana.latterini@unipg.it (L.L.)

**Keywords:** mesenchymal stem cells, stem-cell–nanomaterials interaction, silica star-shaped nanoparticles, gold nanoparticles

## Abstract

The biomedical translational applications of functionalized nanoparticles require comprehensive studies on their effect on human stem cells. Here, we have tested neat star-shaped mesoporous silica nanoparticles (s-MSN) and their chemically functionalized derivates; we examined nanoparticles (NPs) with similar dimensions but different surface chemistry, due to the amino groups grafted on silica nanoparticles (s-MSN-NH_2_), and gold nanoseeds chemically adsorbed on silica nanoparticles (s-MSN-Au). The different samples were dropped on glass coverslips to obtain a homogeneous deposition differing only for NPs’ chemical functionalization and suitable for long-term culture of human Bone Marrow–Mesenchymal stem cells (hBM-MSCs) and Adipose stem cells (hASCs). Our model allowed us to demonstrate that hBM-MSCs and hASCs have comparable growth curves, viability, and canonical Vinculin Focal adhesion spots on functionalized s-MSN-NH_2_ and s-MSN-Au as on neat s-MSN and control systems, but also to show morphological changes on all NP types compared to the control counterparts. The new shape was stem-cell-specific and was maintained on all types of NPs. Compared to the other NPs, s-MSN-Au exerted a small genotoxic effect on both stem cell types, which, however, did not affect the stem cell behavior, likely due to a peculiar stem cell metabolic restoration response.

## 1. Introduction

Tissue engineering is making enormous progress thanks to the rapid development of new synthetic or new assembly methods in material science [1,2,3].

However, despite the prosperous fabrication of novel scaffolds, the production of high-performance materials is still a challenge for tissue regeneration [4]. The ideal scaffold must mimic the natural microenvironment generated by the extracellular matrix (ECM) around cells, including the deposition and architectural organization of ECM components that are necessary for providing physical-chemical cues for cellular adhesion, proliferation, and stem cell differentiation [5,6,7].

Artificial scaffold nanoengineering may offer a breakthrough in tissue regeneration by introducing innovative strategies to enhance the bioactivity and customize the properties of the matrix such as manageable particle size, tunable surface chemistry, biocompatibility, and the large surface-to-volume ratio [8,9,10,11].

In this field, nanoparticles (NPs) are currently one of the main tools explored in materials science, biology, and medicine due to their nanometric size (i.e., less than 100 nm in at least one dimension) and relatively specific manufacturing and functionalization [12,13,14].

Among the wide selection of nanomaterials, mesoporous silica nanoparticles (MSNs) offer many advantages in tissue engineering, including high bioactivity, biocompatibility, and possible biodegradability [15,16,17,18]. These properties have indeed also made MSNs an excellent candidate for biomedical purposes, as in cancer therapy, theranostic applications, and drug delivery [19,20,21]. Moreover, the versatility of silica synthesis guarantees not only a tailored morphology, dimensions, and surface porosity, but also a wide range of surface chemistry, essential for the proper establishment of cell–material interactions [16,22]. Furthermore, the presence of metals in nanoparticles can strongly influence intercellular signaling and cell–cell communication by interacting, for instance, with transmembrane proteins [23]. In particular, gold nanomaterials are appealing due to their high stability, biocompatibility, controlled toxicity, and electronic properties [24,25,26,27]. The preparation of a hierarchical assembly, composed of a silica core and gold nanoparticles on the outer shell [28,29], leads to the manufacturing of hybrid materials with new enhanced features.

Effective tissue engineering requires a suitable stem cell source. In this regard, adult mesenchymal stem cells (MSCs) offer new insights into the progress of therapeutic strategies for different types of diseases (cardiovascular [30] and neurodegenerative [31]) and the development of a biohybrid system for tissue engineering applications [32,33,34]. Multipotent MSCs can differentiate towards osteogenic, adipogenic, chondrogenic, or neural lineages [35,36,37,38,39,40,41,42]. Moreover, through the homing potential, MSCs release different types of bioactive trophic factors involved in molecular events, leading to the repair of damaged tissues, such as modulation of the local immune system, boosting angiogenesis, preventing cell apoptosis, and promoting survival, proliferation rate, and differentiation of stem cells resident in the tissues [43]. These bioactive molecules are also released by MSCs grown on biomaterials and take part in the cross-talk between stem cells and biomaterials [43].

Indeed, the functional interaction of stem cells with a biomaterial is considered one of the main steps for successful tissue engineering application [6,11,44,45]. So far, researchers have focused on evaluating the effect of biomaterials composition and structure of stem cells. In this regard, studies evaluating the direct effect of nanoparticles, and in particular the effect of their functionalization on stem cells are still scarce [7,16,18,46]. Therefore, the development of suitable in vitro models might be useful to establish the effect of NPs characteristics (e.g., size, composition, functionalization, and cargo) on stem cells.

Here, we have synthesized star-shaped mesoporous silica nanoparticles (s-MSN), and s-MSN functionalized with terminal amino groups (s-MSN-NH_2_) and gold nanoseeds-adsorbed silica nanohybrid (s-MSN-Au), and evaluated their effect on human adult Bone Marrow Mesenchymal Stem Cells and Adipose Stem Cells.

To accomplish this aim, we have used an in vitro model consisting of a homogeneous deposition of the above-mentioned nanoparticles suitable for the culture of hBM-MSCs and hASCs and compared their effects in terms of stem cell proliferation, viability, shape and adhesion, and differentiation.

## 2. Materials and Methods

### 2.1. Materials

Tetraethylorthosilicate (TEOS, 98%), (3-aminopropyl) triethoxysilane (APTES, >98%), hexadecyl trimethylammonium p-toluensulfonate (CTATos), triethanolamine (TEAH_3_, >98%), mesitylene (98%), gold(III) chloride trihydrate (HAuCl_4_·3H_2_O, >99.9%), tetrakis(hydroxymethyl)phosphonium chloride solution (THPC, 80% in H_2_O), potassium carbonate (99.99%), formaldehyde solution (37% in H_2_O), hydrochloric acid (HCl, 37%), sodium hydroxide (NaOH, pellets 99%) were all purchased from Sigma–Aldrich (Saint Louis, MO, USA) and used without further purification. Nanopure water from a Millipore Milli-Q gradient system (15.0 MW), methanol (anhydrous, 99.8%), and ethanol (96%) from Sigma–Aldrich were used as solvents.

### 2.2. s-MSN, s-MSN-NH_2_, and s-MSN-Au Production and Characterization

#### 2.2.1. Star-Shaped Mesoporous Silica Nanoparticles (s-MSN) Synthesis

In a three-neck flask equipped with a condenser and a magnetic stirrer, a solution made of CTATos 0.62 g, triethanolamine 100 µL, mesitylene 7.8 mL in 32.5 mL of H_2_O was heated at 80 °C under vigorous stirring for 1 h. Then, 7.8 mL of TEOS were quickly added and the mixture was stirred for an additional 2 h at 80 °C. The solid was collected by centrifugation (3000× *g*, 30 min), and alternatively washed with water (three times) and ethanol (three times). The removal of the surfactant was achieved through acidic treatment. For 100 mg of silica, a mixture of 10 mL of methanol and 100 µL of 37% HCl was added, and the system was kept at 80 °C for 6 h. The nanoparticles were then centrifuged at 3000× *g* for 30 min and washed with water until neutrality of the supernatant was achieved. The last centrifugation was performed in ethanol, and the solid was dried in air at room temperature. This sample is named s-MSN.

#### 2.2.2. Amino Functionalization of Silica Colloids (s-MSN-NH_2_)

Four hundred milligrams of the as-synthesized silica nanoparticles were added to a mixture of 40 mL of ethanol and 10 µL of APTES; the suspension was sonicated for about 10 min and left under stirring for 20 h. The sample s-MSN-NH_2_ was obtained by means of centrifugation (3000× *g*, 30 min), washed with ethanol 4 times, and dried in air at RT (Room Temperature).

#### 2.2.3. Gold Seeds Preparation

Gold seeds were synthesized following the Duff protocol [47], which involves the reduction of Au(III) with tetrakis(hydroxymethyl)phosphonium chloride (THPC). Twelve microliters of THPC were added to a basic solution composed of 46.0 mL of H_2_O and 0.3 mL of NaOH 1 M, and the system was kept under stirring for 30 min; then, 2.0 mL HAuCl_4_ (25.5 mM) was quickly added, obtaining a sudden change of color from yellow to dark brown. After 30 min, gold seeds were ready for adsorption on silica.

#### 2.2.4. Gold Nanoseeds Growth on Silica Surface (s-MSN-Au)

The synthesis of the silica-gold hybrid nanoparticles was carried out through two steps: the adsorption of gold seeds onto s-MSN-NH_2_ and their controlled growth.

The freshly prepared Au seeds (approximately 50 mL) were put in an ultrasound bath, and 25.0 mg of s-MSN-NH_2_ was slowly added (within a time of 15 min). Then, the suspension was stirred for 2 h and the s-MSN-seeds sample was collected by centrifugation (3000× *g*, 30 min) and washed once with water.

Further, 25.0 mg of s-MSN seeds were suspended in 2.5 mL of H_2_O and added to 26.0 mL of an aqueous solution of gold carbonate (prepared with 25.0 mg of potassium carbonate, 100.0 mL H_2_O, and 2.0 mL HAuCl_4_ 25.5 mM). After 30 min of stirring, 200 µL of formaldehyde solution (37% in H_2_O) was added and a color change from brown to dark purple was noted. The particles were centrifuged at 4100 rpm for 20 min, washed with water several times, and dried in an oven at 60 °C, obtaining s-MSN-Au samples.

#### 2.2.5. Characterization of Nanomaterials

The TEM images of the silica-based samples were recorded through a Philips 208 transmission electron microscope with 80 kV of beam acceleration. The size distribution analysis was performed deriving the best Gaussian fit of the experimental data extrapolated through ImageJ software (Rasband, W.S., ImageJ, U. S. National Institutes of Health, Bethesda, MD, USA, http://imagej.nih.gov/ij/, 1997–2016, accessed on 17 March 2021).

The elemental composition and the morphology of s-MSN-Au samples were obtained through a field emission scanning electron microscope (FE-SEM) (FEG LEO 1525) (Zeiss, Oberkochen (Baden-Württemberg), Germany) supporting the energy dispersive X-ray microanalysis (EDX) (Bruker, Billerica, MA, USA).

### 2.3. Human Adult Mesenchymal Stem Cells: Isolation and Culture

Adult Human Bone Marrow-Mesenchymal stem cells (hBM-MSCs) and adult Human Adipose stem cells (hASCs) were isolated and cultured as described in detail in our previous works [22,35,48,49,50,51,52,53]. hBM-MSCs and hASCs were isolated from waste samples (washouts of the medullary cavities of the femur and biopsies, respectively) of donors’ surgery with informed consent. Both procedures were sporadic and carried out in accordance with the Declaration of Helsinki. The mesenchymal phenotype of both hBM-MSCs and hASCs were analyzed by measuring the expression of markers CD45, CD73, CD90, and CD105 (all from BD Biosciences, San Jose, CA, USA) by the flow cytometer FACScan (BD Biosciences, San Jose, CA, USA) and the FlowJo software (Tree Star, Ashland, OR, USA) for data analysis as previously described [48,51].

hBM-MSCs were cultured by plating in culture flasks in DMEM High Glucose (Euroclone S.p.A, Pero (MI), Italy) supplemented with 10% Fetal Bovine Serum (FBS, Euroclone S.p.A, Pero (MI), Italy), 1% penicillin–streptomycin (Euroclone S.p.A, Pero (MI), Italy) and 2 mM L-glutamine (Euroclone S.p.A, Pero (MI), Italy) in a humidified atmosphere and 5% of carbon dioxide (CO_2_) at 37 °C (growth culture medium).

hASCs were cultured by seeding in culture flasks in RPMI-1640 (Euroclone S.p.A, Pero (MI), Italy) added with 10% FBS (Euroclone S.p.A, Pero (MI), Italy), 1% penicillin/streptomycin (Euroclone, Pero (MI), Italy) and 1% L-glutamine (Euroclone, Pero (MI), Italy) at 37 °C, 5% CO_2_ (growth culture medium).

The medium was changed every three days in both stem cell cultures.

### 2.4. Culture of Human Adult Mesenchymal Stem Cells on s-MSN, s-MSN-NH_2_, and s-MSN-Au

#### 2.4.1. Preparation of a Homogeneous Deposition of NPs on Glass Coverslips

A total of 0.5 mg/mL of NPs were resuspended in distilled water and deposited dropwise on sterile glass coverslips, previously placed on a 24-well plate, and dried for 24 h under sterile conditions. All samples were evaluated for the uniformity of the deposition by a light microscope. Further details are reported in the Supplementary file.

#### 2.4.2. Stem Cells Seeding on NPs

The suspension of stem cells (1.5 × 10^3^/mL) was seeded on s-MSN, s-MSN-NH_2_, and s-MSN-Au, respectively, and incubated at 37 °C, 5% CO_2_ for 45 min. Then, the growth culture medium was gently added. Stem cells-nanoparticle cultures were maintained in the growth culture medium and analyzed at different time points for proliferation rate, adhesion, viability, and differentiation toward osteogenic and adipogenic lineages. As a control, the same number of stem cells was seeded on glass coverslips (CTR) and cultured in the growth medium. The medium was changed every three days.

### 2.5. Stem Cell Proliferation on s-MSN, s-MSN-NH_2_, and s-MSN-Au

The growth curve of stem cells on NPs and on CTR was measured at 3, 7, 14, and 21 Days (D). To count the cell nuclei, at each time point, cultures were harvested and fixed in 4% paraformaldehyde and rinsed with PBS, and coverslips were mounted and nuclei were counterstained with Vectashield Antifade Mounting Medium with 4′,6-diamidino-2-phenylindole (DAPI) (Vector Laboratories Inc., Burlingame, CA, USA). The quantification of the number of cells on NPs and CTR was carried out considering 10 different photos (10× magnification) to cover the total NP deposition area, and the DAPI-stained nuclei were counted by using the Cell^f^ software (Soft Imaging System, Olympus, Münster, Germany, version 2.5, Accessed in 2006). Images were acquired using a fluorescence microscope (Eclipse-TE2000-S, Nikon, Tokyo, Japan) equipped with the F-ViewII FireWire camera and Cell^f^ software (Soft Imaging System, Olympus, Münster, Germany, version 2.5, Accessed in 2006). The stem cell proliferation is expressed as a mean of 3 independent experiments ± SEM.

### 2.6. Genotoxic Effects of s-MSN, s-MSN-NH_2_, and s-MSN-Au on Human Adult Mesenchymal Stem Cells

The genotoxic effect was evaluated by the analysis of the DNA double breaks in hBM-MSCs and hASCs, seeded on s-MSN, s-MSN-NH_2_, and s-MSN-Au in growth medium. As positive control, stem cells were treated with 400 µM of H_2_O_2_ (Sigma Aldrich, St. Louis, MI, USA) in growth culture medium for 2 h at 37 °C (CTR + H_2_O_2_). As a reference, stem cells were cultured on glass coverslips (CTR). After washing with PBS and fixing in 4% paraformaldehyde, all samples were analyzed for the expression of phospho-Histone H2AX (pH2AX, Cell signaling Technology, Danvers, MA, USA) through immunofluorescence. After washing with PBS, samples were mounted and nuclei were stained with Vectashield Antifade Mounting Medium with DAPI (Vector Laboratories Inc., Burlingame, CA, USA). The number of pH2AX-positive nuclei and the total number of nuclei in mesenchymal stem cells on NPs and on CTR was counted in 10 different photos (10× magnification) acquired to cover the total NPs deposition. Images were acquired using a fluorescence microscope (Eclipse-TE2000-S, Nikon, Tokyo, Japan) equipped with the F-ViewII FireWire camera and Cell^f^ software (Soft Imaging System, Olympus, Münster, Germany, version 2.5, Accessed in 2006). The results are expressed as mean of three independent experiments± SEM.

### 2.7. Immunofluorescence

Immunostaining was carried out as previously described [7,22,51,54,55]. Briefly, stem cells on NPs and related controls (stem cells on glass coverslips; CTR), after fixing in 4% paraformaldehyde (20 min), were incubated with permeabilization solution (PBS+3% FBS+0.5% Triton X-100) and then in blocking solution (PBS+3% FBS+0.05% Triton X-100) for 1 h at RT each. The F-Actin staining was performed by incubating the samples with Phalloidin (Alexa-fluor-488 phalloidin, Invitrogen™, Grand Island, NY, USA) for 20 min at RT. The immunostaining for anti-βTubulin (Elabscience, Houston, TX, USA), anti-Vinculin (Abcam, Cambridge, UK), anti-Filamin (Santa Cruz Biotechnology, CA, USA) and anti-pH2AX (Cell signaling Technology, Danvers, MA, USA) was performed by incubating the primary human antibody overnight at 4 °C and then with the related secondary antibody conjugated with Alexa-Fluor-594 (Invitrogen™, Grand Island, NY, USA) or Alexa-Fluor-488 (Invitrogen™, Grand Island, NY, USA) for 1 h at RT. Samples were mounted with Vectashield Antifade Mounting Medium with DAPI (Vector Laboratories Inc., Burlingame, CA, USA) and images acquired as above described.

Fluorescent interference of silica-star-shaped NPs and derivatives without cells was evaluated.

### 2.8. Acridine Orange Staining

hBM-MSCs and hASCs on s-MSN, s-MSN-NH_2_, and s-MSN-Au and related controls, were incubated in growth medium with 5 μg/mL Acridine Orange (Sigma-Aldrich, St. Louis, MO, USA) for 15 min at 37 °C. After the incubation, stem cells were washed with PBS three times and mounted in PBS for microscopic analysis. Images were acquired using a fluorescence microscope (Eclipse-TE2000-S, Nikon, Tokyo, Japan) equipped with the F-ViewII FireWire camera (Soft Imaging System, Olympus, Münster, Germany).

### 2.9. Images Quantification of Fluorescence

Fluorescence intensity quantification of Acridine Orange was performed with a custom-made ImageJ script. For each biohybrid system, 50 stained cells were acquired and evaluated. Before quantification, some enhancement operations were performed on each image: (i) non-uniform illumination correction, with a Gaussian smoothing filter, was applied (imflatfield function implemented in MatLab (MathWorks, Inc., Natick, MA, USA, version R2019b)), and (ii) background subtraction was done, applying an opening morphological operation, using a disk of 20-pixel diameter (imopen function implemented in Matlab(MathWorks, Inc., Natick, MA, USA, version R2019b)). Finally, each image was thresholded, cells were masked, and the region properties (integrated density and area) of the masked region of interest (ROI) were calculated for the green and red channels.

A fluorescence intensity projection of the cells was obtained from the relationship:

$$CTCF=cFID-\left(\frac{Ac}{MAb}*MFB\right)$$

where:

*CTCF* = corrected total cell fluorescence
*cFID* = cell fluorescence integrated density
*Ac* = area of masked cell
*MAb* = mean area of background (mean of five different ROI) and
*MFB* = mean fluorescence of the background (mean of five different ROI)

Finally, the quantitative fluorescence analysis of the Acridine Orange staining was calculated as:

$$\frac{G}{R}ratio=\overset{n}{\underset{i=1}{\sum }}\frac{CTCFgreen}{CTCFred}$$

### 2.10. Stem Cell Differentiation

The hBM-MSCs and hASCs on s-MSN, s-MSN-NH_2_, and s-MSN-Au after D21 of culture were evaluated for osteogenic and adipogenic differentiation.

As positive controls of osteogenic and adipogenic differentiation, stem cells were seeded on glass coverslips in 24-well plates, cultured in the growth medium for the first 24 h, and then incubated with a specific medium of differentiation, according to our previous work [50,53].

For the osteogenic differentiation, hMSCs were cultured with differentiation basal medium supplemented with osteogenic SingleQuots (Lonza Walkersville, Inc., Walkersville, MD, USA): dexamethasone, pen/strep, L-glutamine, ascorbate, mesenchymal cell growth supplement (MCGS), and β-glycerophosphate. Cultures were maintained for 21 days in a humidified atmosphere at 37 °C, 5% CO_2_, and the differentiation medium was changed every 3 days.

To achieve adipogenic differentiation, stem cells were subjected to three cycles of induction and maintenance medium (Lonza Walkersville, Inc., Walkersville, MD, USA). Each cycle consisted in stem cells cultured with supplemented adipogenesis induction medium (containing: rh-insulin, l-glutamine, MCGS, dexamethasone, indomethacin, 3-isobuty-lmethylxanthine, penicillin/streptomycin) for 3 days followed by 3 more days of culture in supplemented adipogenic maintenance medium (basal medium supplemented with rh-insulin, l-glutamine, MCGS, penicillin/streptomycin). Cultures were maintained for 21 days in a humidified incubator, 37 °C, 5% CO_2_.

As a negative control, experiments were performed seeding stem cells on glass coverslips in growth medium. All cultures were maintained for 21 days in a humidified incubator at 37 °C, 5% CO_2_.

#### 2.10.1. Alizarin Red

To assess the osteogenic differentiation of stem cells on NPs, Alizarin Red staining (AR) (Sigma-Aldrich, St. Louis, MO, USA) was used. After 21 days, stem cells were washed with PBS twice and fixed with 4% paraformaldehyde for 20 min at RT. Cultures were washed in H2Od and then incubated with 500 μL of Alizarin Red staining (Lonza Walkersville Inc, Walkersville, MD, USA) solution for 20 min at RT. Cells were washed twice with distilled water, and photos were captured with a Canon digital camera (PowerShot G10, Canon, Tokyo, Japan) and brightfield microscopy (Eclipse-TE2000-S, Nikon, Tokyo, Japan).

#### 2.10.2. Oil Red O

To assess adipogenic differentiation of stem cells cultured on NPs, Oil Red O (ORO) (BioVision Inc., Milpitas, CA, USA) stain was used. Stem cells were fixed in 4% paraformaldeheyde for 20 min at RT, washed with PBS, and stained with ORO working solution (ORO 0.3% in isopropanol mixed with H_2_Od (3:2)) for 20 min at RT. Stem cells were then washed in H_2_Od, and images were captured with a Canon digital camera (PowerShot G10, Canon, Tokyo, Japan) and brightfield microscopy (Eclipse-TE2000-S, Nikon, Tokyo, Japan).

### 2.11. Protein Extract and Western Blotting

Cell lysis was carried out as described in our previous works [56,57,58]. Briefly, the cell pellet was resuspended in sodium phosphate buffer (10 mM, pH 6.0), added with 0.1% (*v*/*v*) Nonidet NP40 detergent (Sigma-Aldrich, St. Louis, MO, USA), and sonicated in ice (three rounds of 30 s) using an ultrasonic bath. The NPs were separated from the protein extract by the sample centrifugation at 16,000 rcf for 5′ at 4 °C. The supernatant was collected and protein content measured by the Bradford method [59,60].

Protein extracts were analyzed by SDS-PAGE electrophoresis and subjected to Western blotting as previously reported [7]. We tested, by overnight incubation at 4 °C, the primary antibodies anti-human Vinculin (Abcam, Cambridge, UK), anti-human Superoxide Dismutase 1 (SOD1, Santa Cruz Biotechnology, Dallas, TX, USA), and anti-human Actin (Sigma-Aldrich, St. Louis, MO, USA), followed by incubation for 1 h at RT with anti-rabbit secondary antibody conjugated with horseradish peroxidase (HRP, Cell signaling Technology, Danvers, MA, USA). Immunodetection was performed using the Clarity™ Western ECL Blotting Substrate (Bio-Rad Laboratories S.r.l., Milan, Italy). Densitometric analysis of each immunoblot was carried out using Fiji (Fiji Life-Line version, v.2015, U. S. National Institutes of Health, Bethesda, MD, USA). The results are reported as mean ± SEM of three independent experiments (*p* < 0.05 was considered significant).

### 2.12. Cytomorphometric Measurements

The cytomorphometric descriptors, Cell Aspect Ratio (AR), and Nuclear Shape Index (NSI, commonly known as circularity) were measured on digital images of hASCs and hBM-MSCs on NPs and controls stained with Phalloidin-FITC and DAPI as previously reported. The analysis was performed using Fiji (Fiji Life-Line version, v.2015, U. S. National Institutes of Health, Bethesda, Maryland, USA) [61,62]. NSI = (4π × area)/(perimeter)2. NSI values range from 0 (elongated elliptical morphology) to 1 (circular shape). The AR (ratio of the major axis to the minor axis of the ellipsoid fitted to the cell image) assumes values of 1 for spherical cells and increasingly large values for cells with an elliptical elongated shape.

### 2.13. Statistical Analysis

Data analyses were reported as the mean ± SEM (GraphPad Software, Inc., San Diego, CA, USA, version 4.0). Post-hoc comparison test was performed by the one-way ANOVA and Tukey’s multiple comparison test to determine which means amongst a set of means differ from the rest (GraphPad Software, Inc., San Diego, CA, USA, version 4.0). *p* ≤ 0.05 was considered statistically significant.

## 3. Results

### 3.1. Silica Star-Shaped Nanoparticles: Synthesis, Functionalization, and Au Nanoseeds Growth

**Figure 1:**
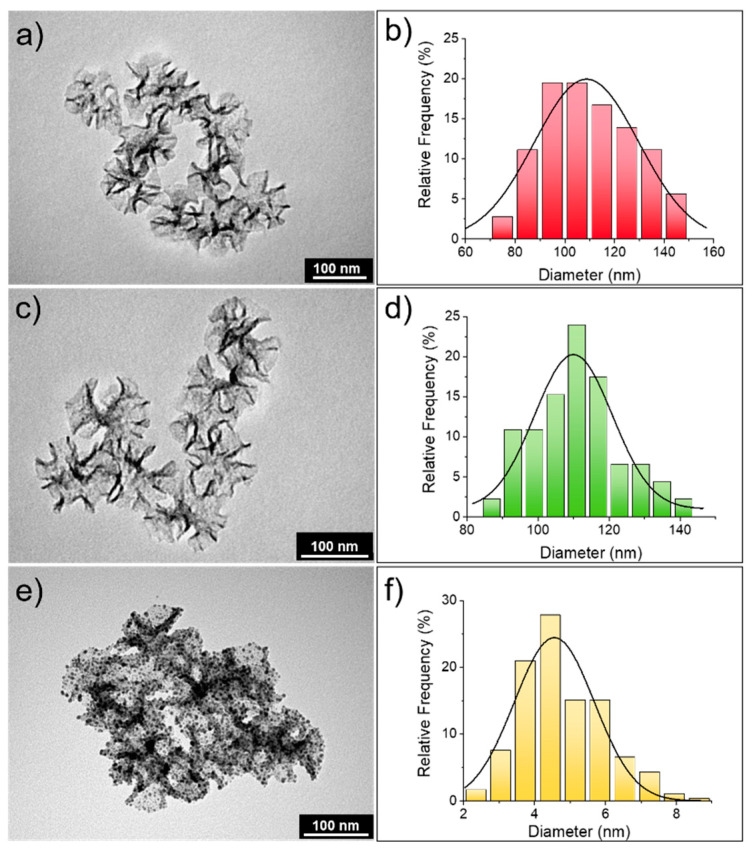
Characterization of star-shaped mesoporous silica nanoparticles (s-MSN), s-MSN-NH_2_, and s-MSN-Au. TEM images (**a**,**c**,**e**) and size distribution obtained from the analysis of TEM images (**b**,**d**,**f**) of s-MSN, s-MSN-NH_2_, and s-MSN-Au samples, respectively (the size distribution in panel f is related to the Au nanostructures adsorbed on silica surface).

Star-shaped mesoporous silica nanoparticles (s-MSN) were synthesized following the procedure presented in the experimental section, wherein the use of a cationic templating agent (CTA^+^), with a counterion presenting high affinity for the polar head of the surfactant, like tosilate (Tos^-^), allowed the formation of nanostructured silica with channel-like porosities [63]. The addition of a swelling agent, such as mesitylene, during the synthesis led to the formation of mesoporous silica with expanded pores [64]; in our case, the opening diameter of the pores ranged between 20 and 25 nm and from the analysis of TEM images (Figure 1a,b), the mean nanoparticle diameter of 109 ± 31 nm was derived. The presence of enlarged surface porosity might enhance the adsorption and interaction with cells.

The grafting of amino groups on the silica surface, using APTES (3-aminopropyl)triethoxysilane), did not alter the morphology and the dimension (110 ± 23 nm) of the particles, as shown in Figure 1c,d. The presence of terminal amino groups is fundamental for the adsorption of Au seeds on the silica surface; indeed, the amino groups can increase the affinity for gold colloids. Moreover, the negative zeta potential of bare silica nanoparticles becomes positive in neat water when terminal amino groups are present on the silica surface [65]. This is a fundamental parameter to take into account, since Au seeds, prepared with Duff protocol [47], present partial negative charges due to hydroxyl groups of the THPC stabilizer.

**Figure 2:**
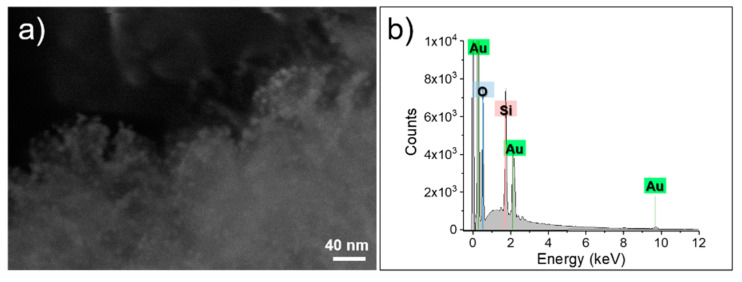
FE-SEM images (**a**) and elemental composition analysis (**b**) of s-MSN-Au sample.

Once Au seeds are electrostatically adsorbed on the silica surface, to obtain their homogeneous growth, the use of a weak reductant is a mandatory requirement. The mild conditions dictated by Au carbonate as precursor and formaldehyde as reducing agent allowed the controlled growth of Au seeds in Au nanostructures, with average dimensions of 4.6 ± 2.1 nm (Figure 1f). As indicated in Figure 1e, the homogeneous coverage of silica particles was fully achieved; this result is also confirmed by FE-SEM analysis (Figure 2a). Moreover, from the EDX analysis (Figure 2b), the presence of gold throughout the s-MSN-Au sample was further demonstrated. Indeed, the peaks located at 1.75 keV and 0.52 keV are attributed to Si and O (K-shell), respectively, clearly derived from the silica component, whereas the peaks at 9.7 keV (L-shell) and 2.1 keV (M-shell) are generated by gold.

### 3.2. s-MSN, s-MSN-NH_2_, and s-MSN-Au and Mesenchymal Stem Cell Behavior and Interaction

**Figure 3:**
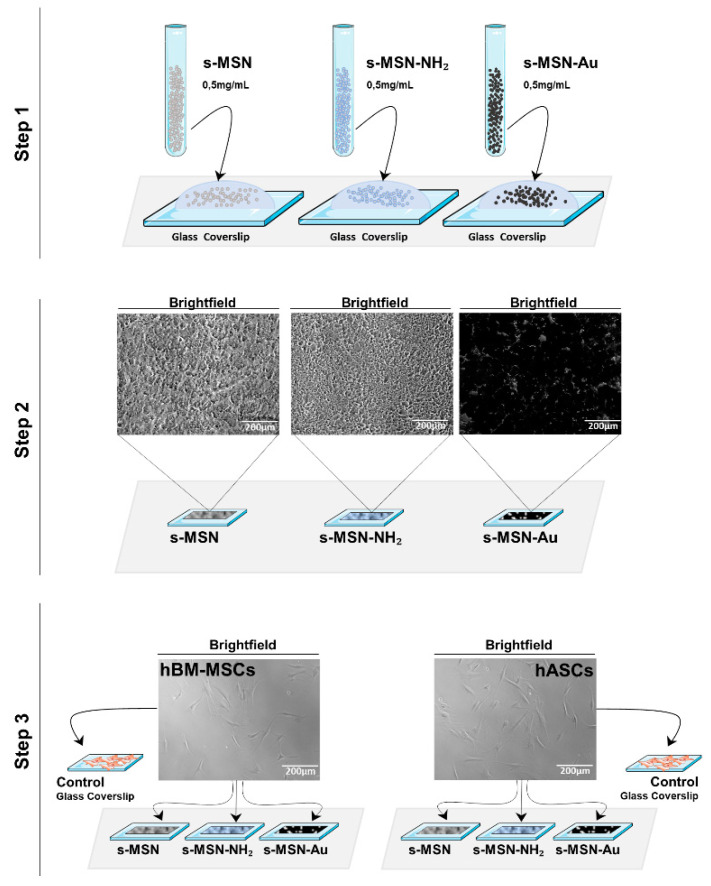
Schematic of the preparation of the homogeneous deposition of s-MSN, s-MSN-NH_2_, and s-MSN-Au on glass coverslips. Dropwise deposition of NPs on glass coverslips (step 1); drying of NPs deposition in a sterile laminar flow hood for 24 h (step 2); seeding of human Bone Marrow–Mesenchymal stem cells (hBM-MSCs) and Adipose stem cells (hASCs) on nanoparticles (NPs) deposition (step 3). Brightfield representative images of NPs deposition after 24 h of its preparation on glass coverslip and brightfield representative images of hASCs and hBM-MSCs used for the study and before the plating on NPs. Scale bar = 200 µm.

First, we generated a suitable in vitro cell-NPs model to study the effect of s-MSN, s-MSN-NH_2_, and s-MSN-Au on human Bone Marrow-Mesenchymal stem cells (hBM-MSCs) and human Adipose stem cells (hASCs). As illustrated in the scheme in Figure 3 (detailed in Supplementary Figures S1 and S2 and in the method section), s-MSN, s-MSN-NH_2_, and s-MSN-Au, in sterile conditions, were dropped on glass coverslips to obtain a homogeneous deposition (step 1) that differ only for the functionalization of above-mentioned NPs. Ninety microliters of each of s-MSN, s-MSN-NH_2_ and s-MSN-Au suspension, necessary to cover the glass coverslips, were dried in a sterile laminar flow hood for 24 h (step 2), and then hASCs and hBM-MSCs were seeded on NPs and maintained in the culture in growth medium (step 3).

**Figure 4:**
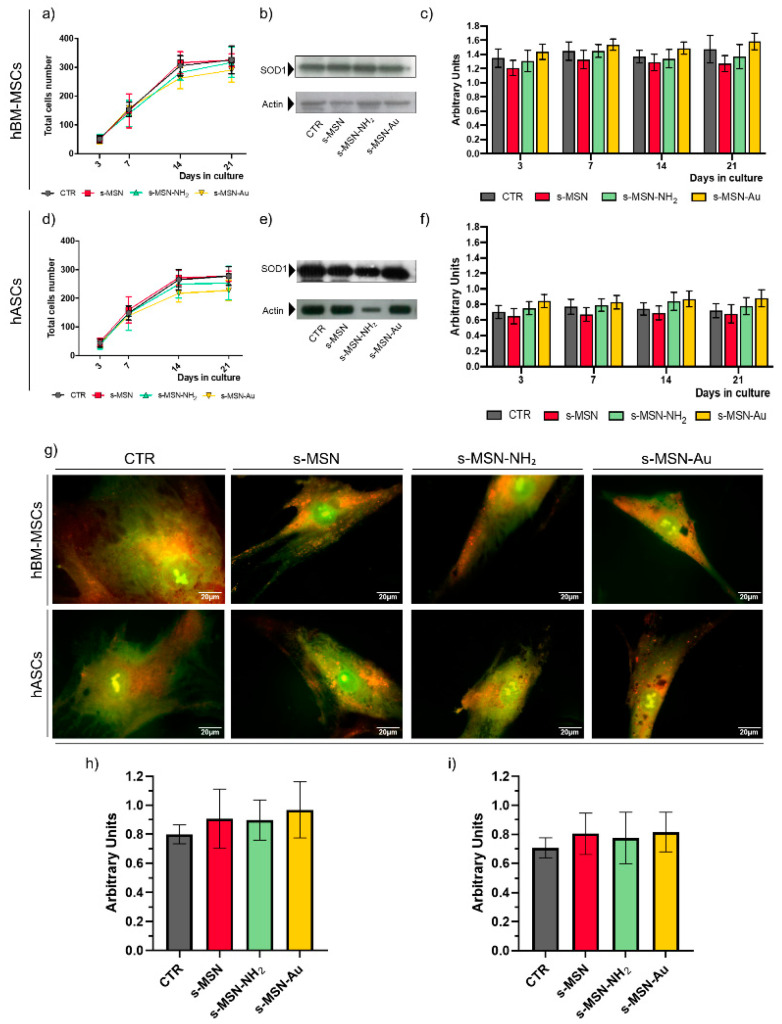
Long-term culture of human adult mesenchymal stem cells on silica star-shaped NPs and functionalized derivatives. (**a**–**c**) hBM-MSCs; (**d**–**f**) hASCs. (**a**,**d**) Proliferation rate. (**b**,**e**) Representative Western blotting bands of SOD1 expression in hBM-MSCs and hASCs at D7, and (**c**,**f**) densitometric analysis of Western blotting performed at each time point of culture. The results of each bar are the mean ± SEM of three independent experiments. *p* < 0.05 was considered significant. (**g**) Representative fluorescence images of Acridine Orange staining. Scale bar = 20 μm. (**h**,**i**) Quantification of the Acridine Orange staining (see method section for details). (**h**) hBM-MSCs; (**i**) hASCs. The results are reported as mean ± SEM of three independent experiments. *p* < 0.05 was considered significant.

**Figure 5:**
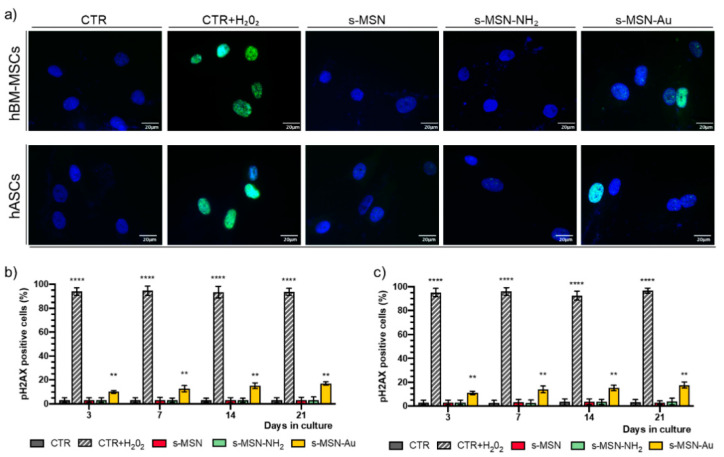
(**a**–**c**) Expression of pH2AX in hBM-MSCs and hASCs. (**a**) Representative immunofluorescence images with anti-pH2AX antibody (Green) and Nuclei (DAPI, blue). Scale bar = 20 μm. (**b**,**c**) Quantification of pH2AX expression in hBM-MSC_S_ (**b**) and hASCs (**c**) performed at each time point of culture. Data are expressed as mean ± SEM, ** *p* < 0.01 **** *p* < 0.0001.

**Figure 6:**
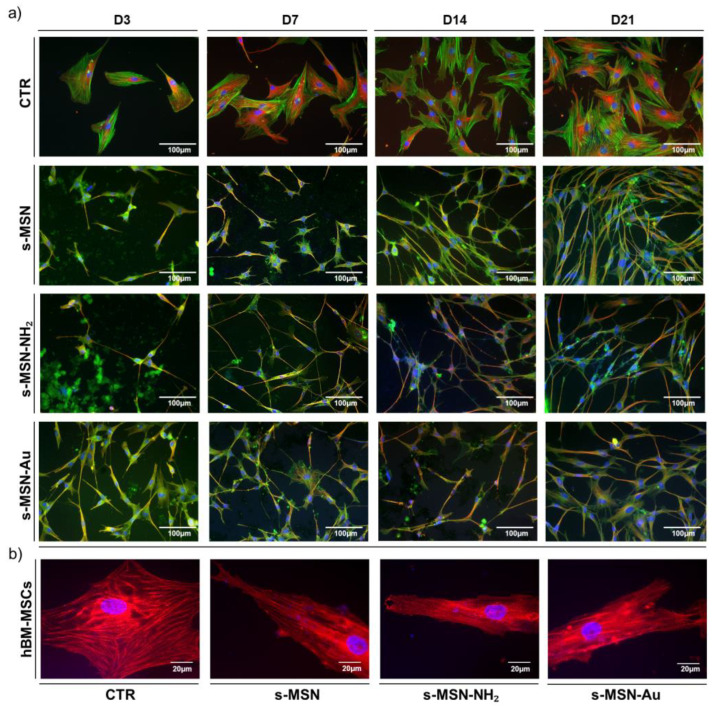
Morphology of hBM-MSCs on s-MSN, s-MSN-NH_2_, and s-MSN-Au during the time in culture. (**a**) Representative images of F-Actin (Phalloidin-Alexa-fluor-488), microtubules (anti-βTubulin antibody, RED), and nuclei (DAPI, blue) on NPs and control system (CTR). Scale bar = 100 μm. (**b**) Representative images of expression of Filamin A (anti-Filamin antibody, RED) and nuclei (DAPI, blue) in stem cells on CTR and s-MSN, s-MSN-NH_2_, and s-MSN-Au. Scale bar = 20 μm.

**Figure 7:**
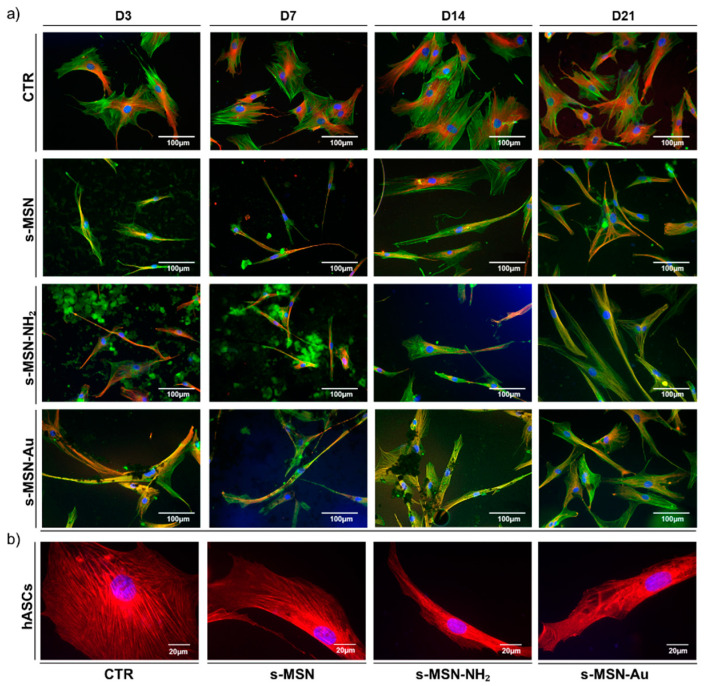
Long-term culture of hASCs morphology on CTR and s-MSN, s-MSN-NH_2_, and s-MSN-Au. (**a**) Representative images of F-Actin (Phalloidin-Alexa-fluor-488, GREEN), Microtubules (anti-βTubulin antibody, RED), and Nuclei (DAPI, blue), Scale bar = 100 μm. (**b**) Representative images of expression of Filamin A (anti-Filamin antibody, RED), and nuclei (DAPI, blue) in stem cells on CTR and s-MSN, s-MSN-NH_2_, and s-MSN-Au. Scale bar = 20 μm.

**Figure 8:**
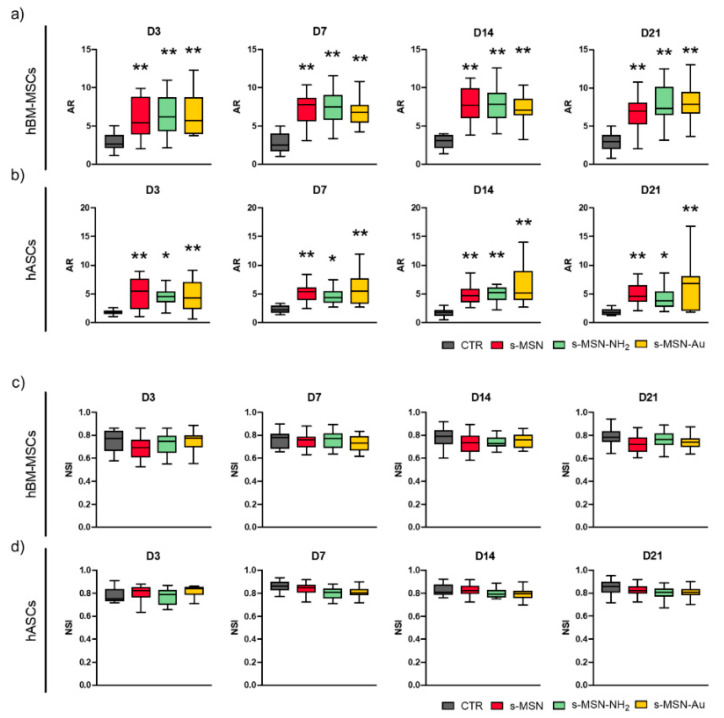
Morphometric measurements of stem cells on NPs deposition.s (**a**,**b**) Aspect ratio (AR) of hBM-MSCs (**a**) and hASCs (**b**) cultured on CTR and s-MSN, s-MSN-NH_2_, and s-MSN-Au. (**c**,**d**) Nuclear Shape Index (NSI) of hBM-MSCs (**c**) and hASCs (**d**) cultured on CTR and s-MSN, s-MSN-NH_2_, and s-MSN-Au. Results are expressed as mean ± SEM. * *p* < 0.05, ** *p* < 0.01.

**Figure 9:**
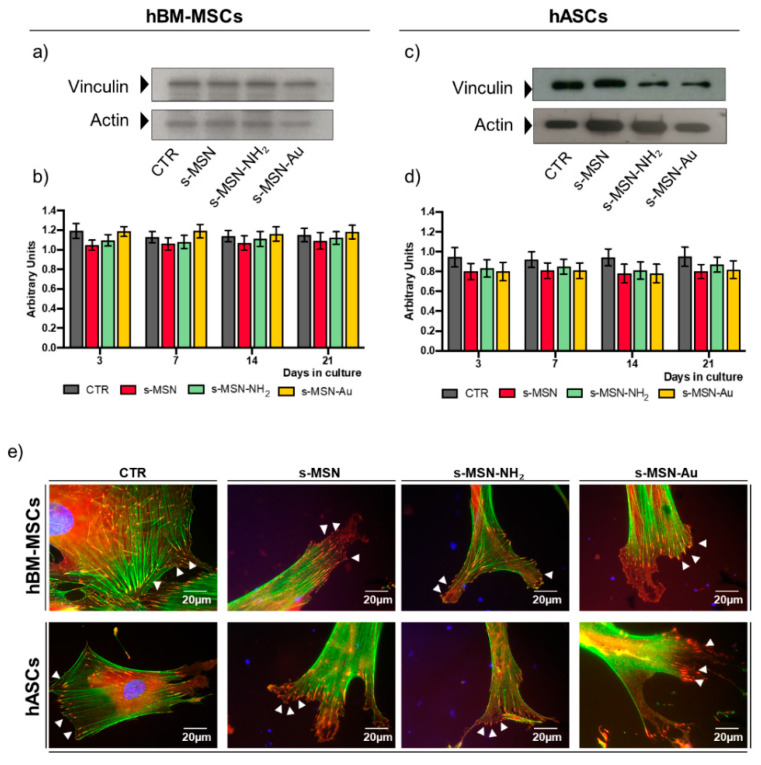
Vinculin expression of both human adult mesenchymal stem cells on s-MSN, s-MSN-NH_2_, and s-MSN-Au. (**a**,**c**) Representative Western blotting bands of Vinculin expression and (**b**,**d**) related quantification by the densitometric analysis at each time point of culture. The results of each bar are the mean ± SEM of three independent experiments. *p* < 0.05 was considered significant. (**e**) Representative images of F-Actin (Phalloidin-Alexa-fluor-488, GREEN) and Vinculin (anti-Vinculin, RED), and Nuclei (DAPI, BLUE). Scale bar = 20 µm.

At different time points, 3, 7, 14 and 21 days (D), cultures were evaluated for the stem cell proliferation rate, viability, shape, adhesion, and differentiation (Figure 4, Figure 5, Figure 6, Figure 7, Figure 8 and Figure 9 and Figures S3–S6). As controls, hASCs and hBM-MSCs were cultured on glass coverslips without NPs deposition in the growth culture medium (Figure 3) and subjected to the same experimental plan (Figure 4, Figure 5, Figure 6, Figure 7, Figure 8 and Figure 9; Figures S2–S6, Supplementary Materials).

#### 3.2.1. Stem Cell Proliferation and Viability

Stem cell proliferation on s-MSN, s-MSN-NH_2_, and s-MSN-Au was monitored by counting the cell number (Figure 4a,d). No statistically significant differences were observed between the growth curve of both hBM-MSCs and hASCs cultured on each type of NP (Figure 4a,d). Growth curves on NPs were also comparable to the growth curves of the respective CTR (Figure 4a,d) and demonstrated the absence of adverse effects of NPs on stem cell proliferation.

Due to the interference of commercial assays with NPs of our system, stem cell viability was determined by evaluating the presence of metabolic alterations and genotoxicity.

We monitored the presence of oxidative stress by evaluating the SOD1 expression [66,67]. Compared to the related controls, no significant statistical differences were detected in the SOD1 expression in hBM-MSCs (Figure 4b,c) and hASCs (Figure 4e,f) on s-MSN, s-MSN-NH_2_, and s-MSN-Au at each time-point analyzed, as demonstrated by the densitometric analyses of Western blotting (Figure 4b,c,e,f).

We evaluated the presence of storage material by using vital staining with Acridine Orange, a lysotropic dye that, in physiological conditions, in a pH-dependent manner, marks in green the cytoplasm and nucleic acid and in orange-red the acidic compartments (e.g., lysosomes, autophagolysosomes) [68,69]. No statistically significant differences were found in the Acridine Orange staining in hBM-MSCs and hASCs on s-MSN, s-MSN-NH_2_, s-MSN-Au, and control counterparts (Figure 4g–i).

We further explored the effect of NPs on stem cell behavior by evaluating the presence of double-strand breaks in DNA through the analysis of the expression of the phosphorylated Histone HA2X (pH2AX) marker. Figure 5 shows the expression of pH2AX in hBM-MSCs and hASCs. After exposure to H_2_O_2_, almost all hBM-MSCs and hASCs highly expressed pH2AX (CTR+H_2_O_2_, positive control), while no expression was observed in stem cells under control culture conditions (CTR) (Figure 5a–c). No expression of pH2AX was observed in hBM-MSCs and hASCs on s-MSN, s-MSN-NH_2_ (Figure 5a–c), whereas a feeble pH2AX expression, accounting for 10–17% positive nuclei with respect to the total number of cell nuclei, was found on s-MSN-Au during the time in culture (Figure 5a–c).

#### 3.2.2. Stem Cell Shape on s-MSN, s-MSN-NH_2_ and s-MSN-Au

The shape of hBM-MSCs and hASCs seeded on s-MSN, s-MSN-NH_2_ and s-MSN-Au was evaluated through the analysis of the architecture of the cytoskeleton by the immune staining of F-Actin fibers and microtubules (Figure 6a, Figure 7a, and Figure 8a,b; Figures S3 and S4, Supplementary Materials). In both systems, the expression of the Filamin A, an F-Actin linking protein, was also analyzed (Figure 6b and Figure 7b).

Results reported in Figure 6 and Figure 7 showed the change of hBM-MSCs and hASCs morphology on s-MSN, s-MSN-NH_2_, and s-MSN-Au compared to related control counterparts.

hBM-MSCs seeded on glass coverslips without deposited NPs maintained the canonical mesenchymal fibroblast-like morphology (Figure 6a; Figure S3, Supplementary Materials) as on tissue culture polystyrene [35,48]. Conversely, hBM-MSCs seeded on s-MSN, s-MSN-NH_2_, and s-MSN-Au displayed a thin elongated morphology as revealed by the organization of Microtubules, F-Actin fibers, and Filamin (Figure 6a,b; Figure S3, Supplementary Materials). Interestingly, the new cytoskeleton architecture allowed the interaction between neighboring stem cells with the formation of a network that was already evidenced on D3, especially on s-MSN-Au, and was maintained over time in culture (Figure 6a; Figure S3, Supplementary Materials). This motif was comparable in hBM-MSCs seeded on all NP types (Figure 6a) and suggests that the morphological change was caused by the basic mesoporous composition of neat NPs.

These shape changes were confirmed by the increase of the cytomorphometric descriptor Aspect Ratio (AR) in hBM-MSCs on s-MSN, s-MSN-NH_2_, and s-MSN-Au with respect to the control (Figure 8a). No statically significant differences were detected in the AR of hBM-MSCs among the three NP types (Figure 8a).

hASCs seeded on glass coverslips also maintained the canonical mesenchymal fibroblast-like shape (Figure 7a; Figure S4, Supplementary Materials), as on tissue culture polystyrene [48]. hASCs cultured on s-MSN, s-MSN-NH_2_, and s-MSN-Au displayed a similar longer shape compared to the control (Figure 7a,b; Figure S4, Supplementary Materials). The new hASC cytoskeleton architecture was comparable on neat and functionalized NPs, as revealed by the staining of F-actin fibers, microtubules, and Filamin A (Figure 7a,b; Figure S4, Supplementary Materials). This structure was confirmed by the measurement of the AR descriptor that was strongly increased in hASCs on s-MSN, s-MSN-NH_2_, and s-MSN-Au compared to the control (Figure 8b). No statically significant differences were detected in the AR of hASCs on neat and functionalized NPs (Figure 8b).

Finally, in both stem cell-NPs systems, we measured the nuclear shape index (NSI, also known as circularity). No statistically significant differences were found in the NSI of hBM-MSCs on all types of NPs and control (Figure 8c). Similarly, comparable NSI values were observed in hASCs on all NP types and control (Figure 8d).

#### 3.2.3. Stem Cell Adhesion on s-MSN, s-MSN-NH_2_, and s-MSN-Au

We further investigated the interaction of hBM-MSCs and hASCs with s-MSN, s-MSN-NH_2_, and s-MSN-Au by evaluating at the molecular level the adhesion of stem cells to neat and functionalized NPs. We analysed the expression of Vinculin, one of the principal constituents of the focal adhesion complex [6], by Western blotting (Figure 9a–d), and the formation of the Viculin focal adhesion spots by immunofluorescence (Figure 9e). There were no significant variations in the expression of Vinculin in both hBM-MSCs and hASCs on s-MSN, s-MSN-NH_2_, and s-MSN-Au compared to related controls (Figure 9a–d). Vinculin adhesion spots appeared well-formed and connected with F-Actin end-terminals in hBM-MSCs and hASCs on neat and functionalized NP types as in control counterparts (Figure 9e, arrows). However, compared to the control, Vinculin adhesion spots allowed the formation of large lamellipodia in both stem cell types on each NP system (Figure 9e, arrows). These structures agreed with the acquisition of the network of hBM-MSCs and with the longer cell morphology of hASCs (Figure 7e).

#### 3.2.4. Stem Cell Differentiation on s-MSN, s-MSN-NH_2_ and s-MSN-Au

Finally, we investigated whether s-MSN, s-MSN-NH_2_, and s-MSN-Au per se were able to induce the differentiation of hBM-MSCs and hASCs. To this end, we analyzed the osteogenic and adipogenic differentiation, two of the differentiation lineages generated by mesenchymal stem cells [50,53]. As controls, hBM-MSCs and hASCs on glass coverslips were cultured in osteogenic or adipogenic medium (see method section for details) or in growth culture medium.

In parallel experiments, hBM-MSCs and hASCs on s-MSN, s-MSN-NH_2_, and s-MSN-Au, after growth in the culture medium for 21 days, were stained with Alizarin Red (staining of osteogenic differentiation) and Oil Red O (staining of adipogenic differentiation). Results, reported in Figures S5 and S6, demonstrated the absence of osteogenic or adipogenic markers in both hBM-MSCs (Figure S5, Supplementary Materials) and hASCs (Figure S6, Supplementary Materials) on each type of NPs, whereas both markers were well expressed on both stem cells after treatment with osteogenic or adipogenic differentiation medium, respectively (Figures S5 and S6, Supplementary Materials).

## 4. Discussion

Nanoparticles are currently considered one of the most powerful tools in nanomedicine applications [14,18,19,24,70,71,72,73,74,75]. Therefore, the research advancement is mainly focused on the improvement of the efficacy of NPs by (i) developing NPs with different biomaterials, different sizes, and different chemical functionalization [46,76,77,78]; (ii) evaluating their biological effects, comparing the naked and functionalized characteristics. These aspects are mandatory for regenerative medicine applications, but, up to now, studies on stem cells and NPs are still scarce and contradictory.

In this work, we presented a comparative study of the effect of neat s-MSN and functionalized s-MSN-NH2 and s-MSN-Au on adult hBM-MSCs and hASCs.

For the purpose of the work, the synthetic protocol used for s-MSN preparation was optimized to obtain mesoporous silica nanoparticles with a star-shaped morphology, enabling us to enhance the surface area of the nanomaterials and to confer a surface roughness that could support the cell adsorption. s-MSN was used to generate functionalized NPs through a postsynthesis chemical modification of the surface; specifically, two additional samples were produced, namely s-MSN-NH_2_ (with terminal amino groups) and s-MSN-Au (gold nanostructures-adsorbed silica nanohybrid). All samples presented a similar dimension, even though the surface chemistry is strongly affected by the different functionalization.

The biological studies were performed in an in vitro model consisting of deposition of s-MSN, s-MSN-NH_2_, and s-MSN-Au on glass coverslips. With our procedure, we obtained a homogenous deposition of NPs on the glass coverslips, which has remained uniform over the whole coverslip area and stable in the culture medium for almost the entire culture time (21 days). In particular, the uniformity of the deposition was almost complete in the first 3 days, the time necessary for the cells to establish the interaction with the substrate; afterward, a small part of nanoparticles was dissolved, but 60–80% of the NPs were still deposited in uniform domains after 21 days in culture. Of note, the procedure did not alter the chemical properties of the neat and functionalized surfaces; therefore, the NPs deposition differed only for the chemical functionalization. These results agree with the work of Andrée L. and co-authors that used mesoporous NPs with different characteristics to obtain a substrate for evaluating the effect on mesenchymal stem cell differentiation [16].

Hence, our model allows the evaluation of the hBM-MSCs and hASC proliferation, shape, adhesion, and differentiation.

We found that the growth curves of hBM-MSCs and hASCs were comparable on neat and functionalized s-MSN and between NPs and control systems. Moreover, all NPs were biocompatible for stem cells, as demonstrated by the absence of metabolic alteration (similar expression of SOD1, an enzyme that is involved in the maintenance of cell physiology due to its detoxifying activity [66,67,79]; similarly, vital Acridine Orange staining, an assay that allows monitoring the presence of storage material within the cells, and the autophagic process [69]). Of note, we observed that 10–17% of hBM-MSCs and hASCs cultured on s-MSN-Au expressed the histone pH2AX, which is a quantitative marker of DNA double-strand breaks caused by different types of physical agents (e.g., ionizing radiation, radiomimetic drugs), chemical agents, and a wide range of genotoxins [80,81]. This result agreed in part with other studies showing the potential cytotoxic effect of gold-shaped NPs [82] but diverged for the low grade of toxicity that we observed in both stem cell types and mostly for the absence of cell proliferation arrest. These differences may be dependent on the chemical composition of NPs synthetized but also on the cell type used in these studies (e.g., type of cell lines; type of stem cells) [82,83,84,85].

Another aspect to be considered is the effect of the interactions of stem cells with NPs. It is well known that the materials, based on their chemical composition and physical properties, are able to induce a specific biological response to the stem cells [7,22,35,36,49,50,51,86]. In fact, stem cells are able to collect the chemical-physical cues of biomaterials and transduce them in intracellular signaling that may influence the cell shape and function [7,22,35,36,49,50,51,86]. In this regard, some research groups have demonstrated that mesoporous NPs may influence cell behavior or stem cell differentiation through their nanoscale structure, functionalization, and delivery system [16,22,87,88].

Confirming these studies, our model offers the advantage of studying the effect of interactions between stem cells and star-shaped mesoporous NPs. We found a different cytoskeleton architecture in hBM- MSCs and hASCs on s-MSN, s-MSN-NH_2_, and s-MSN-Au compared to the control counterparts. The new shape was stem-cell-specific but was the same on all NPs types: (i) thin and elongated shape for hBM-MSCs (AR in NPs systems > AR in CTR) with the formation of network interaction with neighboring cells; (ii) elongated morphology for hASCs (AR in NPs systems > AR in CTR).

Stem cells adhered to NPs by Vinculin focal adhesion spots. These appeared well-formed at the end-terminal of F-actin fibers in both stem cells on s-MSN, s-MSN-NH_2_, and s-MSN-Au as in control systems. However, compared to the control system, on s-MSN, s-MSN-NH_2_, and s-MSN-Au the focal adhesion spot depicted large lamellipodia.

We suggested that the changes of stem cell shape are orchestrated by the mesoporous star-shaped structure of NPs, whereas the different morphology of hBM-MSCs and hASCs on NPs might be the consequence of the cross-talk of each stem cell type characteristics with the NP-material.

As above mentioned, the biomaterial’s chemical–physical property may also influence stem cell differentiation [7,22,35,36,49,50,51,86]. In our system, the change of cell morphology does not correlate with the activation of a specific differentiation program. No osteogenic or adipogenic lineage was found in hBM-MSCs and hASCs on neat s-MSN and functionalized s-MSN-NH_2_ and s-MSN-Au.

## 5. Conclusions

Here, we have presented a comparative study of the effect of functionalized and neat s-MSN on hBM-MSCs and hASCs. We have demonstrated a similar biological effect of functionalized s-MSN-NH_2_ and s-MSN, and a small genotoxic effect of s-MSN-Au on both stem cell types. However, these adverse effects seemed to stimulate a compensatory biological response by hBM-MSCs and hASCs that maintained comparable growth curve, viability, adhesion, and shape as on s-MSN and s-MSN-NH_2_ and control counterparts. Finally, we have highlighted the need for an in vitro model to validate the biological safety of the NPs on stem cells prior to the development of tissue engineering strategies. 

## Data Availability

Data is contained within the article or Supplementary Material.

## Supplementary Materials

The following are available online at https://www.mdpi.com/2079-4991/11/3/779/s1, Figure S1: Time course evaluation of the homogeneous deposition of s-MSN, s-MSN-NH2 and s-MSN-Au on glass coverslips. Scale bar = 200 μm, Figure S2: hBM-MSCs and hASCs on s-MSN, s-MSN-NH2 and s-MSN-Au at D14 of culture. (a) Representative original 8-bit images of F-Actin of hBM-MSCs and hASCs on NPs. (b) Enhanced brightness images of F-Actin of hBM-MSCs and hASCs on NPs. Scale bar = 100 μm, Figure S3: Morphology of hBM-MSCs on s-MSN, s-MSN-NH2 and s-MSN-Au during the culture time (a) Representative images of F-Actin (Phalloidin-Alexa-fluor-488), Microtubules (anti-β-Tubulin antibody, RED), and Nuclei (DAPI, blue) on NPs and control system (CTR) Scale bar = 100 μm, Figure S4: Morphology of hASCs on s-MSN, s-MSN-NH2 and s-MSN-Au during the culture time (a) Representative images of F-Actin (Phalloidin-Alexa-fluor-488), Microtubules (anti-β-Tubulin antibody, RED), and Nuclei (DAPI, blue) on NPs and control system (CTR). Scale bar = 100 μm, Figure S5: Oil Red O staining of hBM-MSCs and hASCs on s-MSN, s-MSN-NH2 and s-MSN-Au at D21. (a) Representative brightfield images of oil red staining of stem cells on glass coverslip cultured in adipogenic differentiation medium (CTR+), and in growth medium (CTR−) (b) of stem cells cultured on each NPs type and (c) of NPs deposition without stem cells. Scale bar = 100 μm, Figure S6: Alizarin red staining of hBM-MSCs and hASCs on s-MSN, s-MSN-NH2 and s-MSN-Au at D21. (a) Representative brightfield images of Alizarin red staining of stem cells on glass coversplip cultured in osteogenic differentiation medium (CTR+), and in growth medium (CTR−) (b) of stem cells cultured on each NPs type and (c) of NPs deposition without the stem cells. Scale bar = 100 μm.
