# Supplementary material for: Functionalized Silica Star-Shaped Nanoparticles and Human Mesenchymal Stem Cells: An In Vitro Model
Source: Nanomaterials (Basel). 2021 Mar 18;11(3):779. doi: 10.3390/nano11030779 (PMC8003255; doi:10.3390/nano11030779)
Supplement: Supplementary file 1 [file nanomaterials-11-00779-s001.pdf]

Supplementary Materials

Functionalized silica star-shaped nanoparticles and human mesenchymal stem cells: an in vitro model

Chiara Argentati[1], Francesco Morena[1], Chiara Fontana[2], Ilaria Tortorella[1], Carla Emiliani[1], Loredana Latterini[2], Giulia Zampini[2]* and Sabata Martino[1]*

[1] Department of Chemistry, Biology and Biotechnology, University of Perugia, Via del Giochetto, 06123; Perugia, Italy; chiara.argentati@unipg.it (C.A.); francesco.morena@unipg.it (F.M.); tortorellailaria@gmail.com (I.T.); carla.emiliani@unipg.it (C.E.)
[2] Department of Chemistry, Biology and Biotechnology, University of Perugia, Via Elce di Sotto, 8, 06123 Perugia, Italy; chiara.fontana@studenti.unipg.it (C.F.); loredana.latterini@unipg.it (L.L.)
* Correspondence: giulia.zampini87@gmail.com (G. Z.); Tel.: +39-075-585-5570 (G.Z.)
* Correspondence: sabata.martino@unipg.it; Tel.: +39-075-585-7442 (S.M.)

Supplementary Materials content:

1. Preparation and stability of the homogeneous deposition of NPs on glass coverslips.
2. Representative images of stem cells on NPs deposition during the time in culture.
3. Immunostaining of the cytoskeleton of of hBM-MSCs or hASCs on s-MSN, s-MSN-NH_2 and s-MSN-Au.
4. Stem cell differentiation on s-MSN, s-MSN-NH_2 and s-MSN-Au

1. Preparation and stability of homogeneous deposition of NPs on glass coverslips.

1.1. Deposition of NPs on glass coverslips and optimization of deposition homogeneity. Different concentration of NPs (1 mg, 0.75 mg, 0.5 mg, 0.25 mg) were resuspended in distilled water and used to obtain a homogeneous deposition on sterile glass coverslips, previously placed on a 24-wells plate. In detail, 90 µL of each NPs suspension was dropwise deposited on glass coverslips and dried for 24h under sterile conditions. The volume was chosen for the ability to cover completely the surface of the glass coverslips. All samples were then evaluated for the uniformity of the deposition by a light microscope. We found that the best concentration of NPs to achieve a homogeneous deposition of nanoparticles was 0.5 mg/mL, that, therefore, was selected for the experiments with stem cells.

1.2. Stability of the NPs deposition. The NPs deposition (0.5 mg/mL of suspension) was monitored for 21 days in culture medium in the cell incubator at 37 °C, 5% CO_2 (Figure S1). Results were comparable for s-MSN, s-MSN-NH_2 and s-MSN-Au. The homogeneous deposition was completely stable at D0 (24 h after deposition and dry, and the time when stem cells were seeded), as well as at D1 and D3 (the time necessary for the establishment of cell-substrate interactions) (**Figure S1**). From D7 to D21the uniformity of the deposition was still in the range 60–80% of the glass coverslips (**Figure S1**).

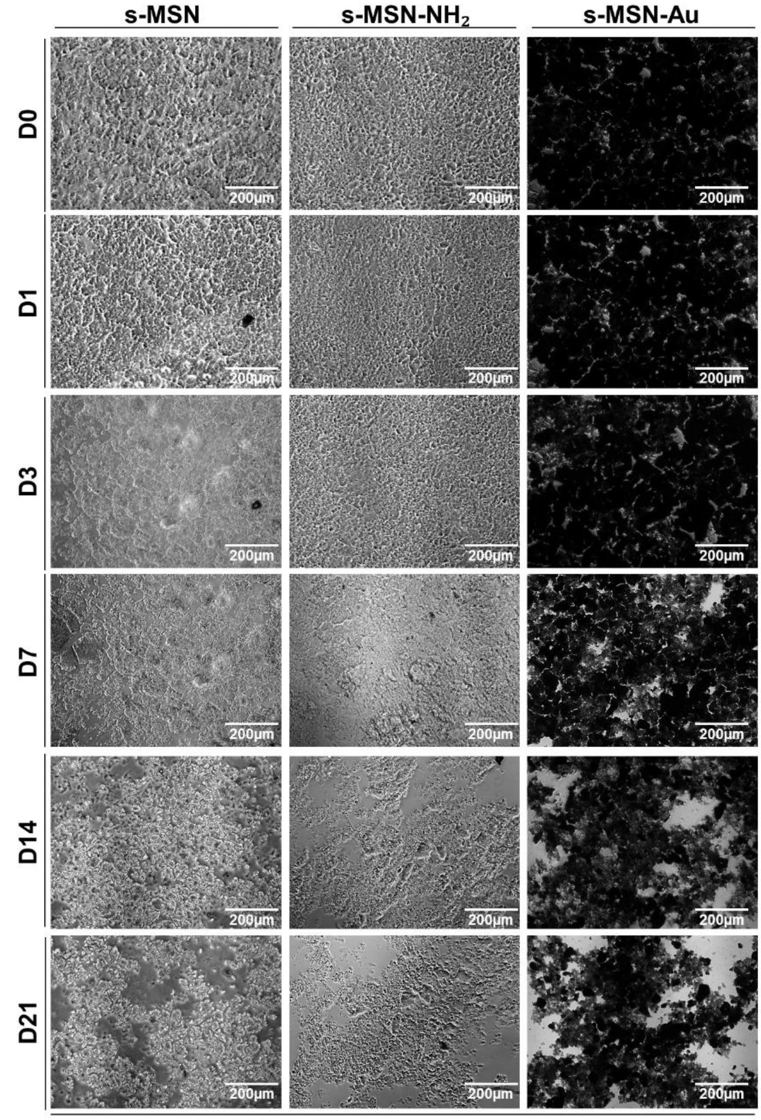

Figure S1. Time course evaluation of the homogeneous deposition of s-MSN, s-MSN-NH_2 and s-MSN-Au on glass coverslips. Scale bar = 200µm.

2. Representative images of stem cells on NPs deposition during the time in culture.

The representative images showed the distribution of stem cells on the NPs deposition. To observe the presence of NPs under cells we enhanced the brightness from images taken in auto mode with fluorescence microscope (Eclipse-TE2000-S, Nikon, To-kyo, Japan) equipped with the F-ViewII FireWire camera (Soft Imaging System, Olympus, Münster, Germany).

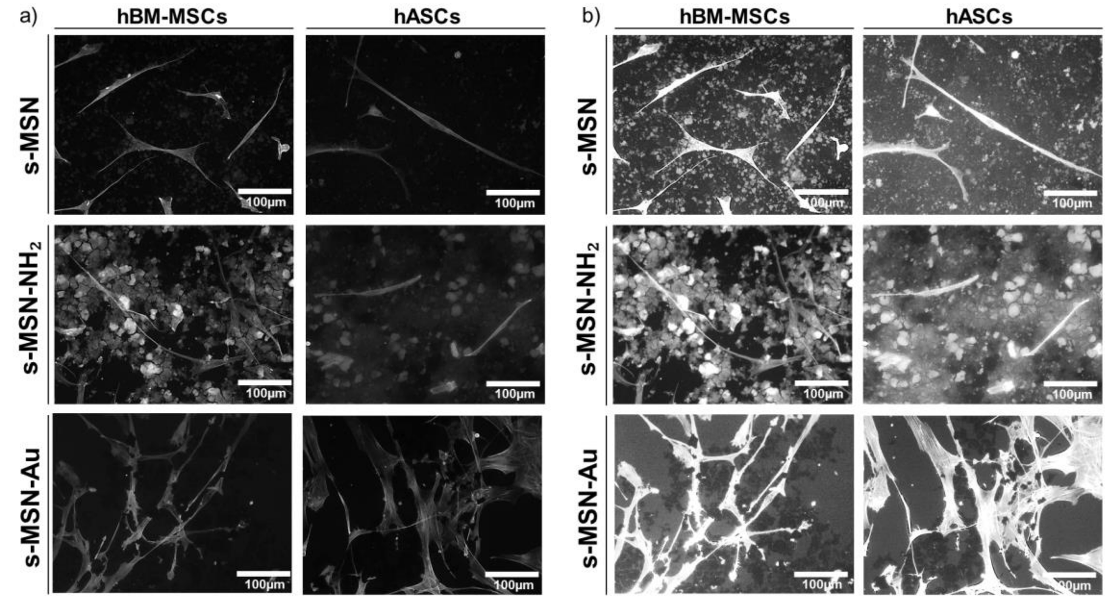

Figure S2. hBM-MSCs and hASCs on s-MSN, s-MSN-NH_2 and s-MSN-Au at D14 of culture. (**a**) Representative original 8-bit images of F-Actin of hBM-MSCs and hASCs on NPs. (**b**) Enhanced brightness images of F-Actin of hBM-MSCs and hASCs on NPs. Scale bar = 100μm.

3. Immunostaining of the cytoskeleton of hBM-MSCs or hASCs on s-MSN, s-MSN-NH2 and s-MSN-Au.

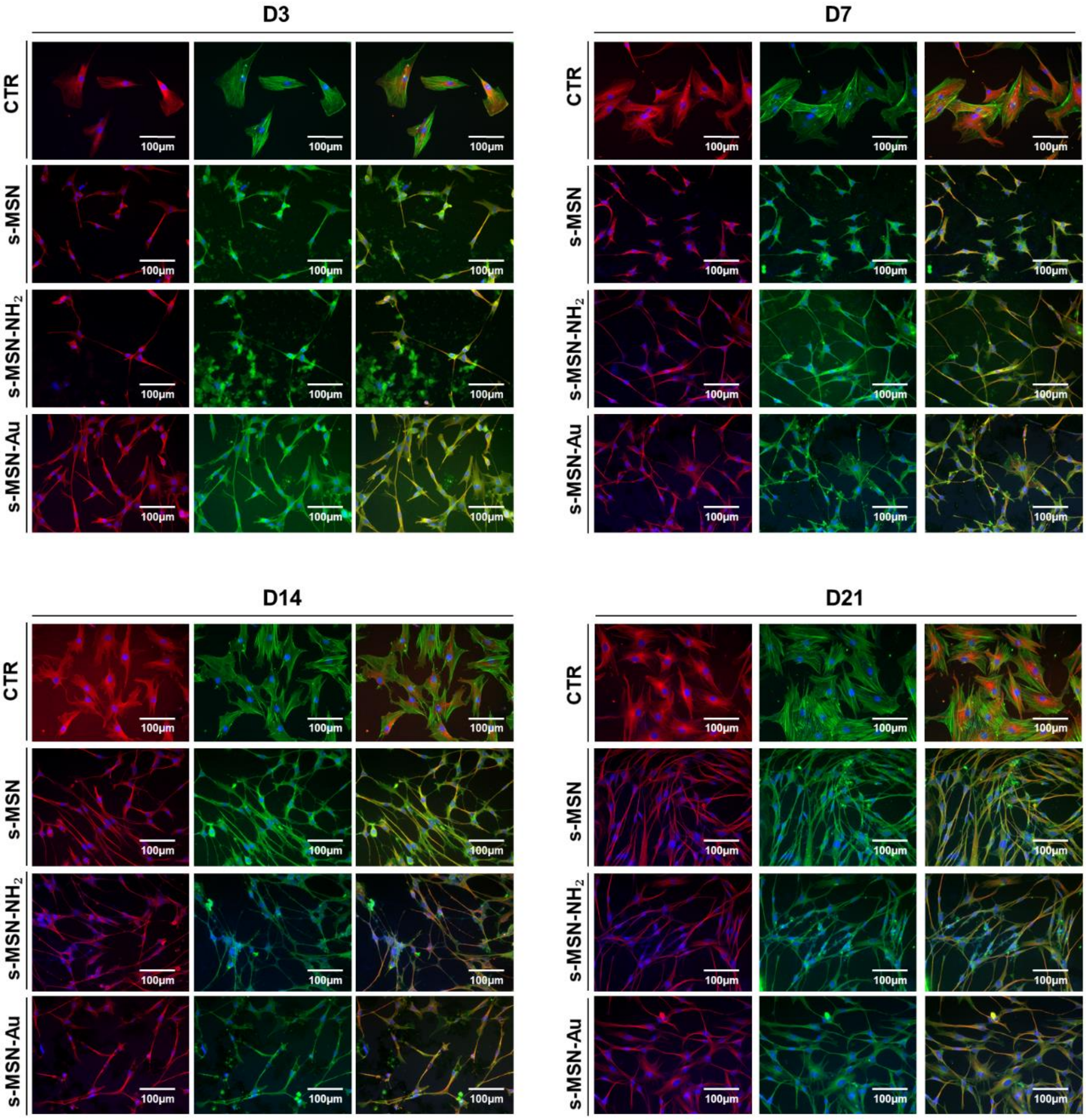

Figure S3. Morphology of hBM-MSCs on s-MSN, s-MSN-NH2 and s-MSN-Au during the culture time (a) Representative images of F-Actin (Phalloidin-Alexa-fluor-488), Microtubules (anti-β-Tubulin antibody, RED), and Nuclei (DAPI, blue) on NPs and control system (CTR) Scale bar = 100µm.

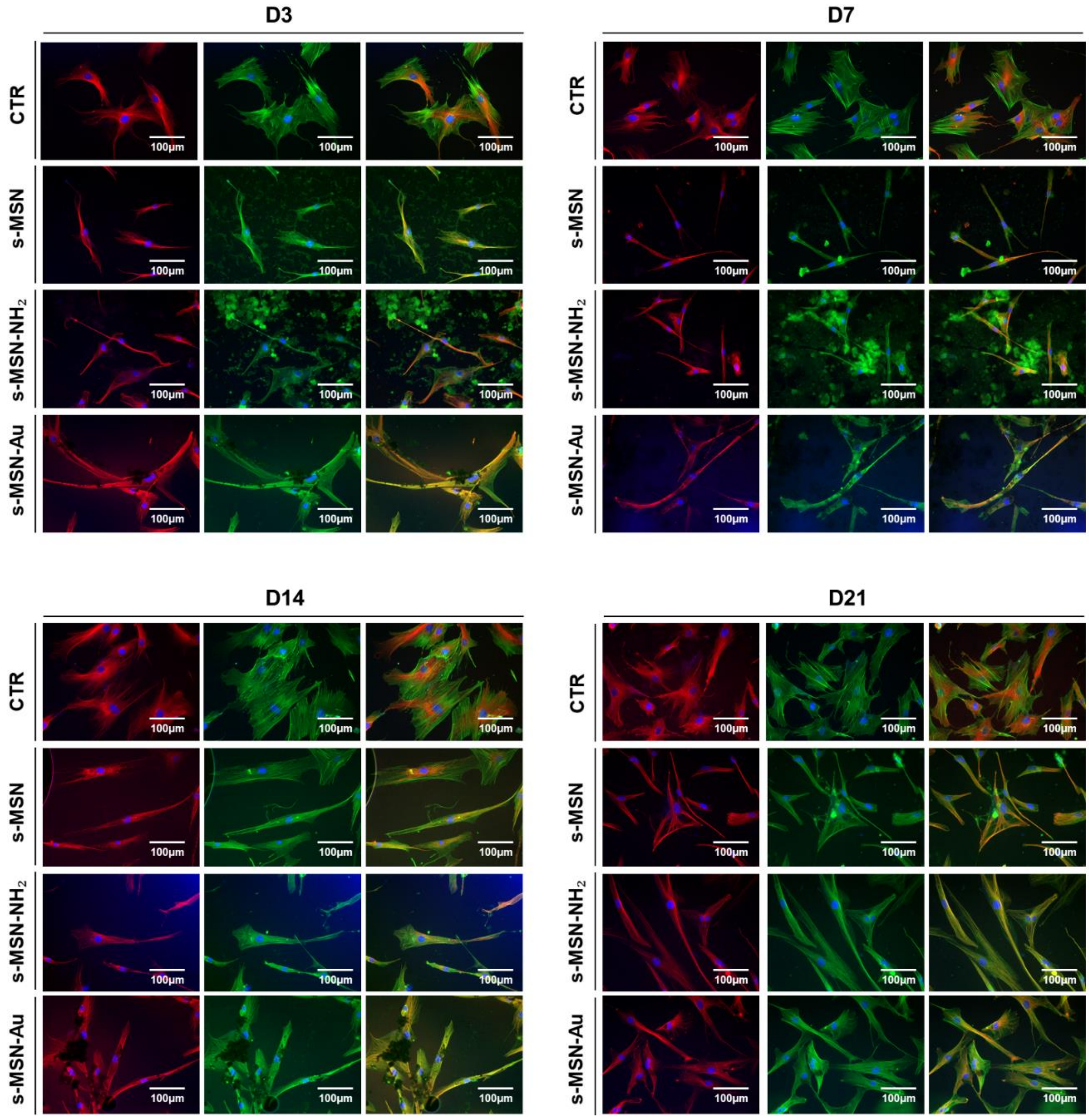

Figure S4. Morphology of hASCs on s-MSN, s-MSN-NH2 and s-MSN-Au during the culture time (a) Representative images of F-Actin (Phalloidin-Alexa-fluor-488), Microtubules (anti-β-Tubulin antibody, RED), and Nuclei (DAPI, blue) on NPs and control system (CTR). Scale bar=100µm.

4. Stem cell differentiation on s-MSN, s-MSN-NH_2 and s-MSN-Au

To evaluate whether stem cells differentiate as a consequence of the interaction with the different NPs deposition used, we analyzed the cell phenotype after 21 days from plating. This time point is necessary for the formation of mature osteocytes and adipocytes following the stem cell differentiation in vitro thanks to the treatment with specific media and inductive factors. To this end, we analyzed the osteogenic and adipogenic differentiation.

4.1. Adipogenic differentiation

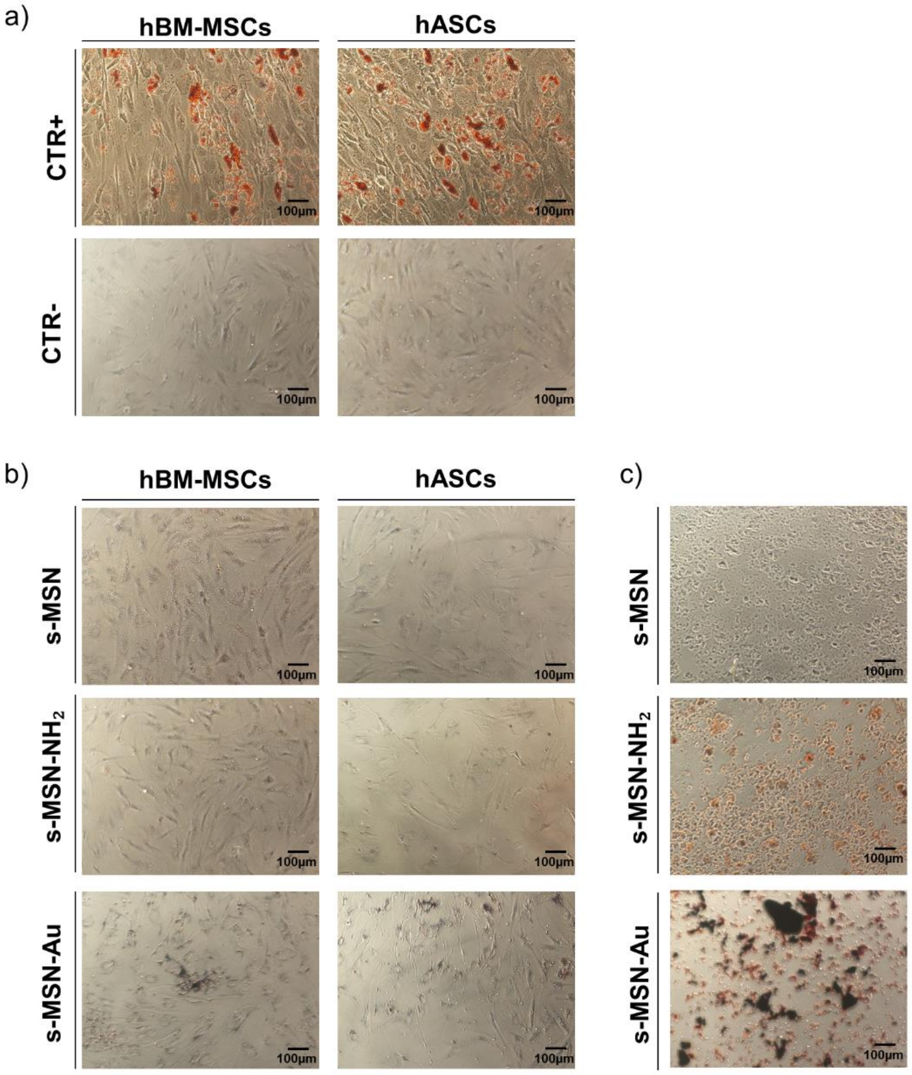

Figure S5. Oil Red O staining of hBM-MSCs and hASCs on s-MSN, s-MSN-NH_2 and s-MSN-Au at D21. (**a**) Representative brightfield images of oil red staining of stem cells on glass coverslip cultured in adipogenic differentiation medium (CTR+), and in growth medium (CTR-) (**b**) of stem cells cultured on each NPs type and (**c**) of NPs deposition without stem cells. Scale bar = 100μm.

4.2. Osteogenic differentiation

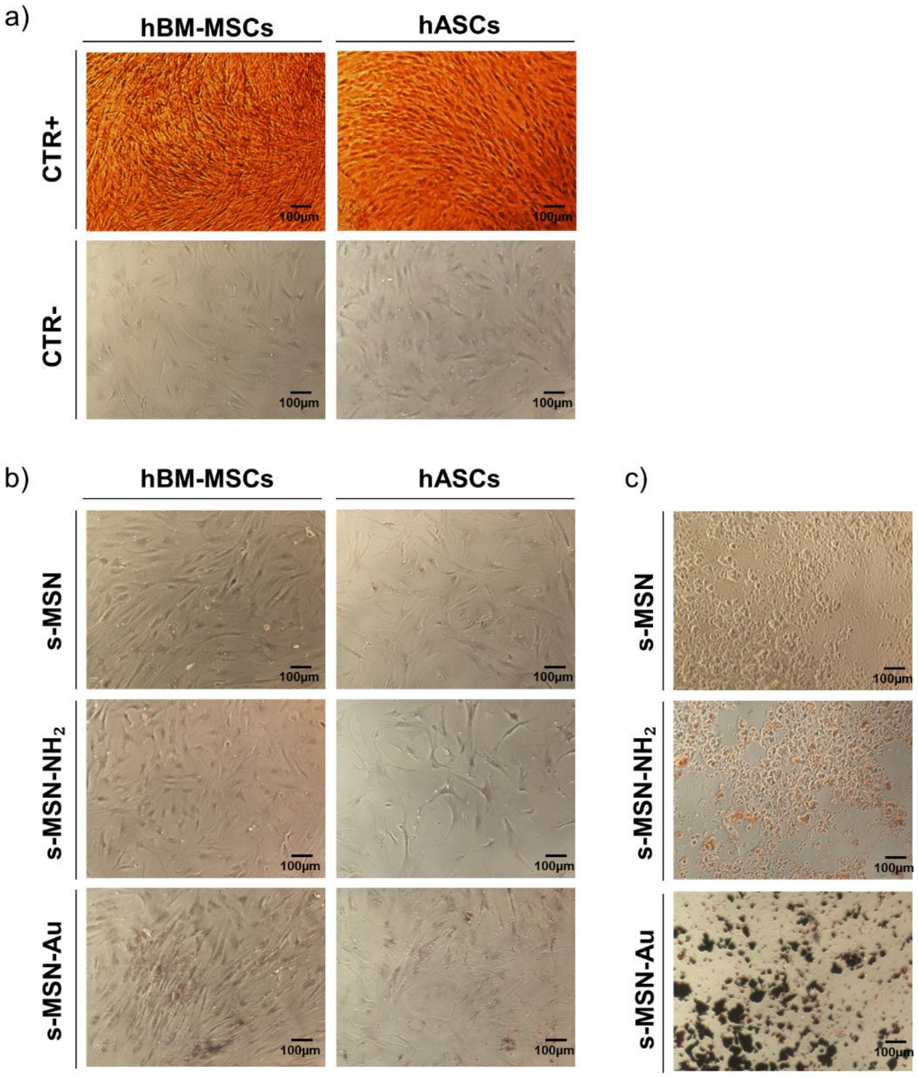

Figure S6. Alizarin red staining of hBM-MSCs and hASCs on s-MSN, s-MSN-NH2 and s-MSN-Au at D21. (**a**) Representative brightfield images of Alizarin red staining of stem cells on glass coversplip cultured in osteogenic differentiation medium (CTR+), and in growth medium (CTR-) (**b**) of stem cells cultured on each NPs type and (**c**) of NPs deposition without the stem cells. Scale bar = 100µm.
